# Editorial: Highlights in psychology: social anxiety

**DOI:** 10.3389/fpsyg.2024.1404923

**Published:** 2024-04-26

**Authors:** Anastassia Zabrodskaja, Antonios Dakanalis

**Affiliations:** ^1^Baltic Film, Media and Arts School, Tallinn University, Tallinn, Estonia; ^2^Department of Medicine and Surgery, University of Milano Bicocca, Monza, Italy

**Keywords:** social anxiety (SA), cognition, relationships, mental health, depression, cultural differences, emotions, neurobiology

The aim of the Research Topic is to provide a comprehensive overview of the current research landscape surrounding social anxiety. Social anxiety is a pervasive mental health condition characterized by intense fear and discomfort in social situations, often leading to significant impairment in various areas of life such as relationships, work, and school.

Through this edition, the goal is to shed light on various aspects of social anxiety, including its cognitive, emotional, interpersonal, and cultural dimensions. The Research Topic seeks to showcase a diverse range of research methodologies and perspectives within the field of psychology, encompassing disciplines such as Personality and Social Psychology, Clinical Psychology, and Cognition.

The Research Topic delves into various specific themes, spanning errors in cognition like hypermentalizing and their correlation with social anxiety, along with exploring the repercussions of social anxiety on diverse relationship dynamics encompassing familial, romantic, professional, and platonic spheres. Additionally, it scrutinizes the comorbidity nexus between social anxiety and other mental health afflictions like depression and eating disorders, whilst also examining social anxiety across age demographics, from children to adolescents and young adults. The discourse extends to encompass assessment and treatment methodologies tailored for social anxiety, considering cultural dimensions including prevalence, manifestation, and treatment paradigms across different societies. Moreover, it investigates gender disparities and cultural influences on social anxiety, underlining the role of sociocultural factors in its formulation. Furthermore, it elucidates the intricate interplay between emotions, notably shame, and social anxiety, alongside delving into the neurobiological and psychophysiological underpinnings of this phenomenon.

The Research Topic contributes to our understanding of social anxiety and provide insights that can inform both theory and practice in psychology. This Research Topic includes articles that focus on social anxiety, demonstrating the wide range of research conducted in the field of Psychology, including areas such as Personality and Social Psychology, Clinical Psychology, and Cognition. Key conclusions drawn from the articles include the interdisciplinary nature of studying social anxiety, the introduction of concepts like “Alexinomia”, and the exploration of its relationships with other psychological factors such as olfactory reference disorder and childhood maltreatment. The role of personality traits, cultural influences, and technological advancements like social media are also highlighted, alongside the impact of current events such as the COVID-19 pandemic on social anxiety.

Articles within this Research Topic use methodologies from Personality and Social Psychology, Clinical Psychology, Cognition, and other related fields, highlighting the interdisciplinary nature of studying social anxiety. Several articles delve into the relationship between social anxiety and other disorders or conditions, such as olfactory reference disorder, childhood maltreatment, substance use disorders, and cognitive processing differences. This highlights the importance of understanding how social anxiety interacts with and may be influenced by other psychological factors.

The interplay between personality traits and social anxiety is a recurring theme, emphasizing the significance of individual differences in shaping the experience and expression of social anxiety. Cultural influences, such as self-construals among Chinese individuals, and technological advancements, such as social media use, are shown to have implications for social anxiety. These findings underscore the importance of considering cultural and technological contexts in understanding and addressing social anxiety.

Current events, such as the COVID-19 pandemic, can have significant implications for social anxiety and related behaviors. Understanding how contextual factors influence social anxiety is crucial for developing effective interventions and treatments. The exploration of therapeutic approaches, such as dialectical behavior therapy skills groups, suggests promising avenues for intervention in treating social anxiety disorder. Identifying effective treatment modalities is essential for improving outcomes for individuals with social anxiety.

This collection of articles enhances our comprehension of social anxiety across various domains, from its underlying mechanisms to its impact on individuals' lives, and explores potential avenues for intervention and treatment. Articles explore various aspects of social anxiety, including its interaction with different disorders, cognitive processes, technological influences, and cultural contexts. They also propose therapeutic approaches such as dialectical behavior therapy skills groups, aiming to improve interventions and treatments for social anxiety disorder. Each article contributes uniquely to the growing body of knowledge, shedding light on different aspects such as cognitive processing, cultural influences, therapeutic interventions, and the interplay with other psychological factors.

Ditye et al. introduce the concept of a specific fear related to social interaction.

Reuter et al. explore the relationship between specific disorders or conditions and social anxiety.

Okano and Nomura move into examining specific aspects of social anxiety and its interaction with other psychological factors.

Macovei et al. continue exploring the interplay between personality traits and social anxiety.

Liu et al. expand the discussion to include the influence of childhood experiences, cultural factors, and substance use disorders on social anxiety.

Zhu et al. shift focus to how social anxiety affects cognitive processes, particularly in interpreting non-verbal cues.

Yang et al. examine the relationship between social media use and social anxiety, adding a technological and cultural dimension to the discussion.

Thériault et al. explore the impact of social expectations and feedback on individuals with social anxiety.

Bagheri et al. offer a data-driven approach to understanding social dysfunction and its predictors, adding empirical evidence to the discussion.

Xia et al. consider the impact of current events (such as the COVID-19 pandemic) on social anxiety and related behaviors, incorporating relevant contextual factors.

Villalongo Andino et al. conclude by exploring potential therapeutic approaches for addressing social anxiety, suggesting avenues for intervention and treatment.

To conclude, the Research Topic deepens our understanding of social anxiety across multiple domains, offering insights into its mechanisms, impact on individuals' lives, and potential avenues for intervention and treatment.

## Author contributions

AZ: Conceptualization, Data curation, Formal analysis, Funding acquisition, Investigation, Methodology, Project administration, Resources, Software, Supervision, Validation, Visualization, Writing—original draft, Writing—review & editing. AD: Writing—review & editing.

